# Dysregulated expression of circular RNAs serve as diagnostic and prognostic markers in ovarian and cervical cancer: A PRISMA-compliant systematic review and meta-analysis

**DOI:** 10.1097/MD.0000000000027352

**Published:** 2021-10-01

**Authors:** Fengyuan Liu, Xinrui Wu, Huixia Zhu, Feng Wang

**Affiliations:** a Department of Clinical Medicine, Medical School of Nantong University, Nantong, Jiangsu, China; b Department of Laboratory Medicine, Public Health School of Nantong University, Nantong, Jiangsu, China; c Department of Laboratory Medicine, Affiliated Hospital of Nantong University, Nantong, Jiangsu Province, China.

**Keywords:** cervical cancer, CircRNA, diagnostic, ovarian cancer, prognostic

## Abstract

**Introduction:**

Recent studies have reported a connection between non-coding RNAs such as circular RNAs (circRNAs) and the prognosis of various cancers. However, the mechanism of circRNA in ovarian cancer and cervical cancer has not been consistent. We evaluated the diagnostic and prognostic roles of circRNAs in ovarian and cervical cancer by meta-analysis.

**Methods:**

Pooled hazard ratios with 95% confidence intervals were to estimate overall survival. Diagnostic efficacy was estimated by sensitivity, specificity and area under curve.

**Results:**

By searching PubMed, Embase, the Web of Science databases, and other sources, we obtained a total of 22 studies with 2059 patients from Asia population. High expression levels of oncogenic circRNAs were significantly associated with poor prognoses both in ovarian and cervical cancer. However, elevated expression levels of tumor-suppressor circRNAs were linked with favorable survival time in ovarian cancer. As for diagnostic role, the area under the curve value in ovarian cancer and cervical cancer is 0.89 and 0.93, respectively.

**Conclusions:**

CircRNAs have the prospect of becoming a promising biomarker for diagnosis and prognosis of ovarian and cervical cancer. Accordingly, circRNAs might be novel indicators and targets of therapy for ovarian and cervical cancer.

## 1 Introduction

Ovarian and cervical cancer are the 2 most common tumors in females.^[1]^ Among the tumor-related causes of death in young women, cervical cancer ranks second.^[1]^ Approximately 527,600 new cases and 265,700 mortalities annually and showing an increasing trend with cervical cancer.^[2]^ Besides, ovarian cancer was the 7th most common cancer in 2012 and it was predicted that the number of deaths due to ovarian cancer will increase to 254,000 in 2035 by the Globocan study.^[3,4]^

CircRNAs are a special kind of endogenous noncoding RNAs that have connected 3′ and 5′ ends that form a closed covalent ring structure through the cyclization of exons or introns. They are also competitive RNAs that, along with long-chain non-coding RNAs, coregulate microRNAs.^[5–8]^ CircRNA participates in the growth and development of cancer, diabetes, nervous system disorders, cardiovascular diseases, and other diseases through various biological roles, such as sponge action, protein translation, and binding protein action.^[9–12]^ In recent years, more and more researchers have found that circRNA plays an important role in the development of ovarian and cervical cancer. However, no consistent results have been obtained regarding the mechanism of circRNA in the 2 cancers.^[13–26]^

Through the study of Zhao et al,^[27]^ oncogenic human papillomaviruses (HPVs) produce circRNA, which inhibits cancer cell growth both in vitro and in tumor xenografts. And, Guan et al^[28]^ reported circPUM1 promoted tumorigenesis and progression of ovarian cancer through sponge miR-615–5p and miR-6753-5p. Enhanced understanding of the role of circRNAs in ovarian and cervical cancer survival will provide more accurate prognostic information and could improve clinical decision-making in trial design and cancer treatment. Accordingly, we conducted this meta-analysis based on plenty of original documents to identify the role of circRNAs in ovarian and cervical cancer.

## 2 Methods

### 2.1 Ethics statement

All analyses were based on previously published studies, this article does not contain any studies with human participants or animals performed by any of the authors; thus, ethical approval and patient consent are not applicable.

### 2.2 Search strategy

Based on the guidelines of the Meta-analysis of Observational Studies in Epidemiology (MOOSE) group and Preferred Reporting Items for Systematic Reviews and Meta-analysis (PRISMA) statement,^[29]^ we searched the Web of Science, EMBASE, PubMed, Cochrane library and CNKI databases up to August 1, 2020. The searching items were: “circRNA,” “circular RNA,” “hsa circ,” “gynecological cancer,” “ovarian cancer,” “oophoroma,” “carcinoma of the ovary,” “metrocarcinoma,” and “cervical cancer.” To avoid missing documents, we manually screened the reference lists of the retrieved articles.

### 2.3 Eligibility criteria

Eligible articles conformed to the following criteria: the subjects were ovarian cancer or cervical patients confirmed by histopathological diagnosis and the clinical data were complete; the article evaluated the relationship between circRNA expression, clinicopathological features, diagnosis and prognosis; and (3) it was a case-control study. The exclusion criteria were: the subjects of the study were not human; the publication was not a primary research publication (eg, a review, correspondence, repeated publication, conference summary), there were no data available in the article.

### 2.4 Quality assessment

The quality of primary diagnostic accuracy studies was assessed by the QUADAS-2 tool. The QUADAS tool consists of 4 key domains, including patient selection, index test, reference standard, and flow of patients. The answer to risk for bias could be rated as “no” (0 points), “yes” (1 point), or “unclear” (0 points).^[30]^ The Newcastle-Ottawa Scale was used to evaluate the quality of case-control studies from three aspects: selection, comparability, and results.^[31]^ Publications that were rated <6 points were considered of low quality; high quality was >6 points.

### 2.5 Data extraction

Two researchers (LFY, WXR) separately evaluated the suitability of all retrieved studies and extracted the relevant data. The 2 researchers contacted a third researcher (WF) when there was a disagreement. The following data were extracted: title, first author, ethnicity, year, sample type, patient size, circRNA signature, follow-up (months), TNM stage, expression status, detection methods, sensitivity (SEN), specificity (SPE), cutoff value setting, pooled hazard ratios (HRs), overall survival (OS), disease-free survival, and their corresponding 95% confidence intervals (CIs). When HRs and 95% CIs could not be extracted directly, we applied the methods described by Parmar et al^[32]^ and Tierney et al^[33]^ to estimate the values from the Kaplan–Meier curves in the articles.

### 2.6 Statistical analysis

HRs and 95% CIs were used to estimate OS. Sensitivity, SPE, and area under the curve (AUC) were involved in the diagnostic meta-analysis. Heterogeneity was assessed by the *χ*^2^ test and expressed by the *I*^2^ index and was judged to be significant if the *I*^2^ value was >50%. We used SEN analyses to investigate potential sources of heterogeneity. Publication bias was evaluated quantitatively using Deeks funnel plot, Begg tests, and Egger tests. Statistical analyses were performed using Revman 5.3 and Stata 15.1 software (Stata Corporation, College Station, TX).

## 3 Results

### 3.1 Selection of studies

**Figure 1:**
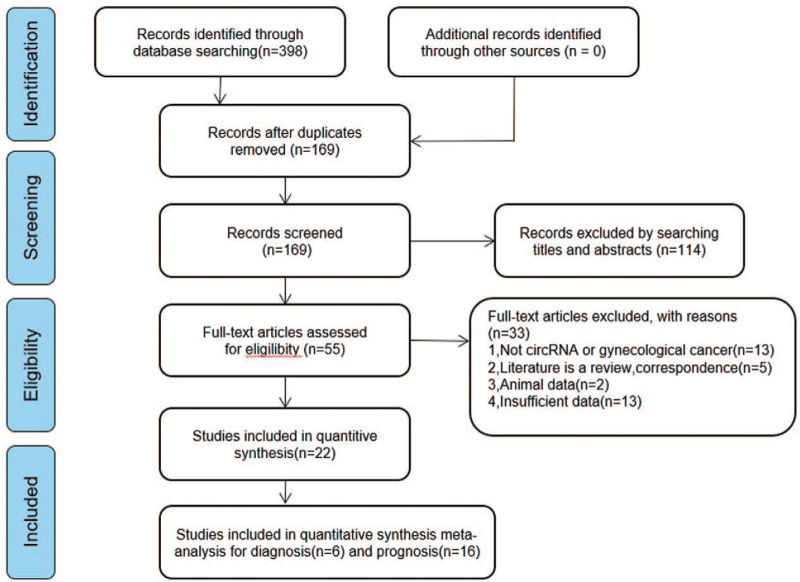
Flow diagram of the study selection process.

A total of 398 articles were initially obtained from the databases and other sources based on keywords (Fig. 1). Among these articles, 229 duplicated articles were removed, and 169 articles remained. By looking through titles and abstracts, 55 articles were left for further full-text review. We then reviewed the full texts of these articles carefully and excluded an additional 33 articles. One article has 2 studies independently.^[34]^ Finally, 22 studies (20 articles)^[13–26,34–39]^ were included in this meta-analysis, including 6 for diagnosis,^[34–38]^ and 16 for prognosis.^[13–26,34,39]^

### 3.2 Characteristics of included studies and quality assessment

The study characteristics are shown in Tables 1 and 2. A total of 2059 patients with ovarian and cervical cancer from Asia were collected from the 22 included studies.^[13–26,34–39]^ The article incorporated two types of gynecological tumors including ovarian tumors and cervical tumors. The publication years ranged from 2017 to 2020. The follow-up period varied from 48 to 110 months. With the QUADAS-II criteria, the scores of all diagnostic researches were ≥4 (see Fig. 1, Supplemental Content, which illustrates the Study quality assessed by the QUADAS II tool). Assessed by the Newcastle-Ottawa Scale, the points of the prognostic trials were ≥6 (see Table 3, Supplemental Content, which illustrates the study quality assessed via the Newcastle-Ottawa Scale checklist.). The results indicated that all of the articles are of high quality.

**Table 1:** Main characteristics of studies for diagnosis analysis.

				Sample size				Diagnostic power		
Study	Year	circRNA signature	Cancer type	Case	Control	Detection methods	Expression status	Sen	Spe	AUC
Wang et al^[38]^	2017	hsa_circ_0101996	Cervical cancer	87	55	qRT-PCR	Upregulated	0.897	0.836	0.906
Wang et al^[38]^	2017	hsa_circ_0101119	Cervical cancer	87	55	qRT-PCR	Upregulated	0.701	0.927	0.887
He et al^[34]^	2020	circ_0018289	Cervical cancer	96	96	qRT-PCR	Upregulated	0.807	0.896	0.907
Pei et al^[36]^	2020	hsa_circ_0013958	Ovarian cancer	45	45	qRT-PCR	Upregulated	0.800	0.911	0.912
Wang et al^[37]^	2019	circSETDB1	Ovarian cancer	32	28	qRT-PCR	Upregulated	0.783	0.733	0.83
Hu et al^[35]^	2019	circBNC2	Ovarian cancer	83	83	qRT-PCR	Downregulated	0.952	0.855	0.923

AUC = area under the ROC curve, qRT-PCR = quantitative real-time polymerase chain reaction, sen = sensitivity, spe = specificity.

**Table 2:** Main characteristics of studies for prognosis analysis.

Study	Ethnicity	Year	Sample type	Patient size	CircRNA signature	Follow-up, mo	Cancer type	Expression status	Survival	OS HR (95% CI)	Detection methods
Xu et al^[19]^	Asian	2020	tissue	60	Circ0004390	50	Ovarian cancer	Upregulated	OS	2.55 (1.02–6.41)	qRT-PCR
Liu et al^[39]^	Asian	2018	tissue	69	circHIPK3	80	Ovarian cancer	Upregulated	OS/DFS	2.18 (1.06–3.78)	qRT-PCR
Luo et al^[17]^	Asian	2018	tissue	77	Circ-ITCH	50	Ovarian cancer	Downregulated	OS	0.20 (0.06–0.65)	qRT-PCR
Sun et al^[18]^	Asian	2019	tissue	25	circPIP5K1A	70	Ovarian cancer	Upregulated	OS	2.03 (0.41–10.02)	qRT-PCR
Zhang et al^[20]^	Asian	2019	tissue	86	CircPLEKHM3	110	Ovarian cancer	Downregulated	OS	0.38 (0.16–0.94)	qRT-PCR
Sheng et al^[13]^	Asian	2019	tissue	24	circUBAP2	80	Ovarian cancer	Upregulated	OS	2.39 (1.04–5.46)	qRT-PCR
Li et al^[16]^	Asian	2020	tissue	60	CircRNA_100395	67	Ovarian cancer	Downregulated	OS	0.37 (0.17–0.79)	qRT-PCR
Chen et al^[14]^	Asian	2019	tissue	103	Circ-ABCB10	60	Ovarian cancer	Upregulated	OS	2.99 (1.29–6.94)	qRT-PCR
Zhang et al^[21]^	Asian	2019	tissue	33	hsa_circ_0051240	60	Ovarian cancer	Upregulated	OS	2.21 (0.59–8.24)	qRT-PCR
Zou et al^[22]^	Asian	2018	tissue	78	circLARP4	70	Ovarian cancer	Downregulated	OS/DFS	0.35 (0.20–0.66)	qRT-PCR
Sun et al^[26]^	Asian	2019	tissue	54	circ–FAM53B	60	Ovarian cancer	Upregulated	OS	2.18 (1.18–4.05)	qRT-PCR
He et al^[34]^	Asian	2020	tissue	192	circ_0018289	48	Cervical cancer	Upregulated	OS	2.61 (1.51–4.51)	qRT-PCR
Ji et al^[15]^	Asian	2019	tissue	35	circSLC26A4	50	Cervical cancer	Upregulated	OS	2.53 (0.81–7.89)	qRT-PCR
Ding et al^[23]^	Asian	2019	tissue	46	Circ-ATP8A2	60	Cervical cancer	Upregulated	OS	2.32 (1.03–5.23)	qRT-PCR
Song et al^[25]^	Asian	2018	serum	39	hsa_circRNA_101996	80	Cervical cancer	Upregulated	OS	1.69 (0.43–6.68)	qRT-PCR
Hong et al^[24]^	Asian	2019	tissue	48	circCLK3	70	Cervical cancer	Upregulated	OS	4.09 (1.03–16.19)	qRT-PCR

CI = confidence interval, DFS = disease-free survival, HR = hazard ratio, OS = overall survival, qRT-PCR = quantitative real-time polymerase chain reaction.

### 3.3 Functions and mechanisms of circRNA in ovarian and cervical cancer

#### 3.3.1 CircRNA in ovarian cancer

The expression and mechanisms of circRNAs in ovarian cancer are presented in Table 3. Several circRNAs (circ0004390, circHIPK3, circPIP5K1A, circUBAP2, circ-ABCB10, hsa_circ_0051240, circ FAM53B) exert their oncogenic roles in ovarian cancer cells.^[13,14,18,19,21,26,39]^ For instance, circPIP5K1A acts as a sponge of miR-661 to promote ovarian cancer progression via regulation of IGFBP5. Nevertheless, Various circRNAs (such as circ-ITCH, circPLEKHM3, circRNA_100395, circLARP4) have been found to inhibit the proliferation of ovarian cancer cells by acting as miRNA sponges.^[16,17,20,22]^ CircPLEKHM3 suppresses the proliferation and migration of ovarian cancer cells by sponging miR-9 to regulate BRCA1, DNAJB6, and KLF4.

**Table 3:** The expression and mechanisms of circRNAs in cervical cancer and ovarian cancer.

Cancer type	CircRNA signature	Expression	Function	Mechanism	Reference
Ovarian cancer	circ0004390	Upregulated	Cancer oncogene	Regulates ovarian cancer proliferation by miR-198/MET axis	^[19]^
Ovarian cancer	circHIPK3	Upregulated	Cancer oncogene	N/A	^[39]^
Ovarian cancer	circ-ITCH	Downregulated	Cancer suppressor gene	Sponges miR-10a-α	^[17]^
Ovarian cancer	circPIP5K1A	Upregulated	Cancer oncogene	Downregulation of IGFBP5 triggered by induction of increased miR-66 levels	^[18]^
Ovarian cancer	circPLEKHM3	Downregulated	Cancer suppressor gene	Inactivates the PI3K/AKT and Wnt/β-catenin pathways via promoting BRCA1, DNAJB6a and KLF4 expression by sponging miR-9	^[20]^
Ovarian cancer	circUBAP2	Upregulated	Cancer oncogene	Increases CDH2 expression by sponging miR-144	^[13]^
Ovarian cancer	circRNA_100395	Downregulated	Cancer suppressor gene	Increases E-cadherin expression and reduces N-cadherin and Snail expression via promoting p53 expression by sponging miR-1228	^[16]^
Ovarian cancer	circ-ABCB10	Upregulated	Cancer oncogene	Inhibits miR-1271, miR-1252 and miR-203 expression	^[14]^
Ovarian cancer	hsa_circ_0051240	Upregulated	Cancer oncogene	Inhibits the miR-637/KLK4 axis	^[21]^
Ovarian cancer	circLARP4	Downregulated	Cancer suppressor gene	N/A	^[22]^
Ovarian cancer	circ–FAM53B	Upregulated	Cancer oncogene	Sponges the miR-646/VAMP2 and miR-647/MDM2 signaling pathways	^[26]^
Cervical cancer	circ_0018289	Upregulated	Cancer oncogene	N/A	^[34]^
Cervical cancer	circSLC26A4	Upregulated	Cancer oncogene	Increases HOXA7 expression by sponging miR-1287-5p	^[15]^
Cervical cancer	circ-ATP8A2	Upregulated	Cancer oncogene	Increases EGFR expression by sponging miR-433	^[23]^
Cervical cancer	hsa_circRNA_101996	Upregulated	Cancer oncogene	Inhibits of miR-8075	^[25]^
Cervical cancer	circCLK3	Upregulated	Cancer oncogene	Sponges miR-320a	^[24]^

N/A = not applicable.

#### 3.3.2 CircRNA in cervical cancer

The included studies^[15,23–25,34]^ showed that the circRNA-miRNA-mRNA regulatory networks made key functions in managing cervical cancer oncogenesis. Various circRNAs (such as circSLC26A4, circ-ATP8A2) promoted the oncogenesis of cervical cancer by increasing the downstream mRNAs, including HOXA1 and EGFR via sponging miRNAs^[15,23]^ (Table 3). In addition, overexpression of circRNA induces cancer cell invasion and metastasis by activating the molecular pathway. For example, circCLK3 enhanced cell proliferation, migration and invasion through sponging miR-320a and promoting FoxM1 expression.^[24]^

### 3.4 Prognostic value of circRNA on ovarian and cervical cancer

#### 3.4.1 Ovarian cancer studies

**Figure 2:**
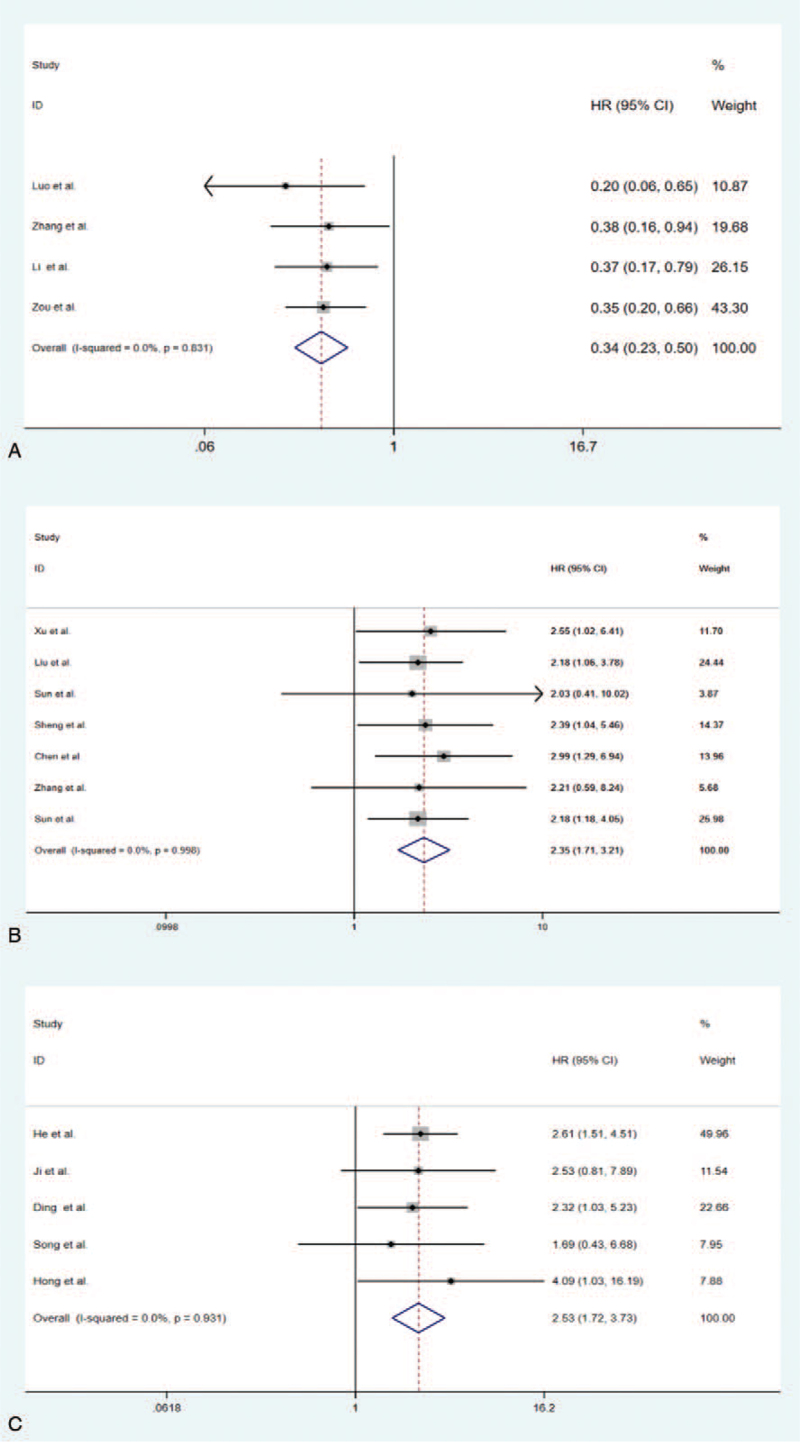
(A) Forest plots for overall survival according to the type of Ovarian cancer tumor-suppressor circRNA. (B) Forest plots for overall survival according to the type of Ovarian cancer oncogenic circRNA. (C) Forest plots for overall survival according to the type of cervical cancer oncogenic circRNA.

Eleven studies were included in the meta-analysis of ovarian cancer.^[13–24,32,37–39]^ Elevated expression of tumor suppressor circRNAs was related to a favorable prognosis (HR = 0.34, 95% CI: 0.23–0.50, *P* < .001) (Fig. 2 A). A fixed-effect model was applied because there was low heterogeneity (*I*^2^ = 0%, *P* = .831). Conversely, high expression of tumor-promoter circRNAs was linked with an unfavorable prognosis (HR = 2.35, 95% CI: 1.71–3.21, *P* < .001) (Fig. 2 B). There was no significant heterogeneity (*I*^2^ = 0%, *P* = 1.000), so the fixed-effect model was performed for this analysis as well.

#### 3.4.2 Cervical cancer studies

Five studies were included in the analysis of the effect of circRNA on ovarian cancer overall survival. The results showed that high expression of Cervical cancer tumor-promoter circRNAs was associated with poor survival time (HR = 2.53, 95% CI: 1.72–3.73, *P* < .001) (Fig. 2 C). No significant heterogeneity was found across the studies (*I*^2^ = 0%, *P* = .931). The relation between tumor suppressor circRNA and overall survival failed to obtain due to the lack of suitable original studies.

### 3.5 Diagnosis analysis of circRNA on ovarian and cervical cancer

#### 3.5.1 Ovarian cancer studies

**Figure 3:**
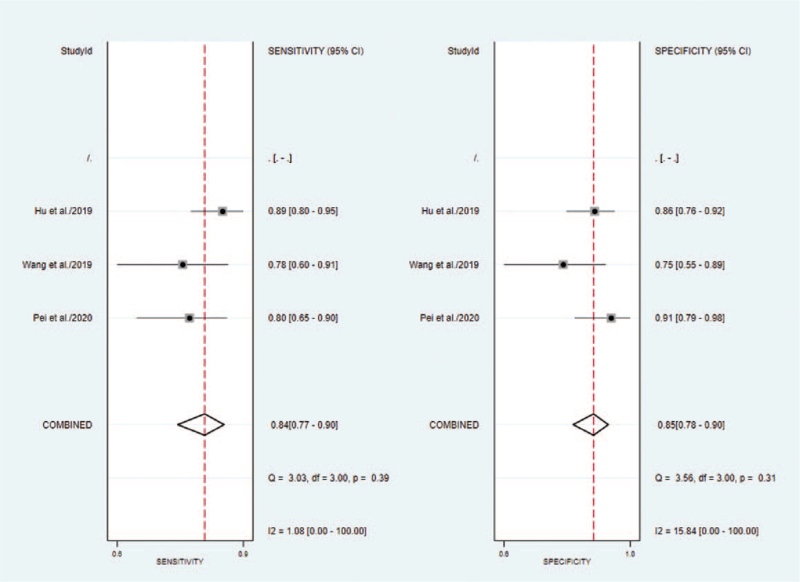
Forest plot of sensitivity and specificity of circRNAs for the diagnosis of Ovarian cancer.

The outcomes of pooled SEN and SPE of ovarian cancer were shown in Figure 3. A fixed-effect model was applied due to the no significant heterogeneity between the groups. The summary estimates are as follows: SPE, 0.85 (95% CI 0.78–0.90); SEN, 0.84 (95% CI 0.77–0.90); besides, a summary receiver operator characteristic curve was carried out in Figure 5 and AUC was 0.89 (95% CI 0.86–0.92).

#### 3.5.2 Cervical cancer studies

**Figure 4:**
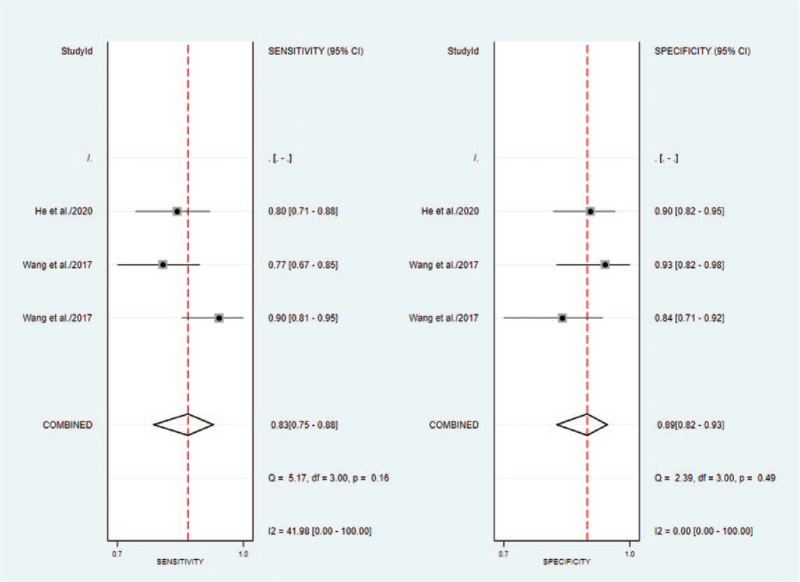
Forest plot of sensitivity and specificity of circRNAs for the diagnosis of Cervical cancer.

**Figure 5:**
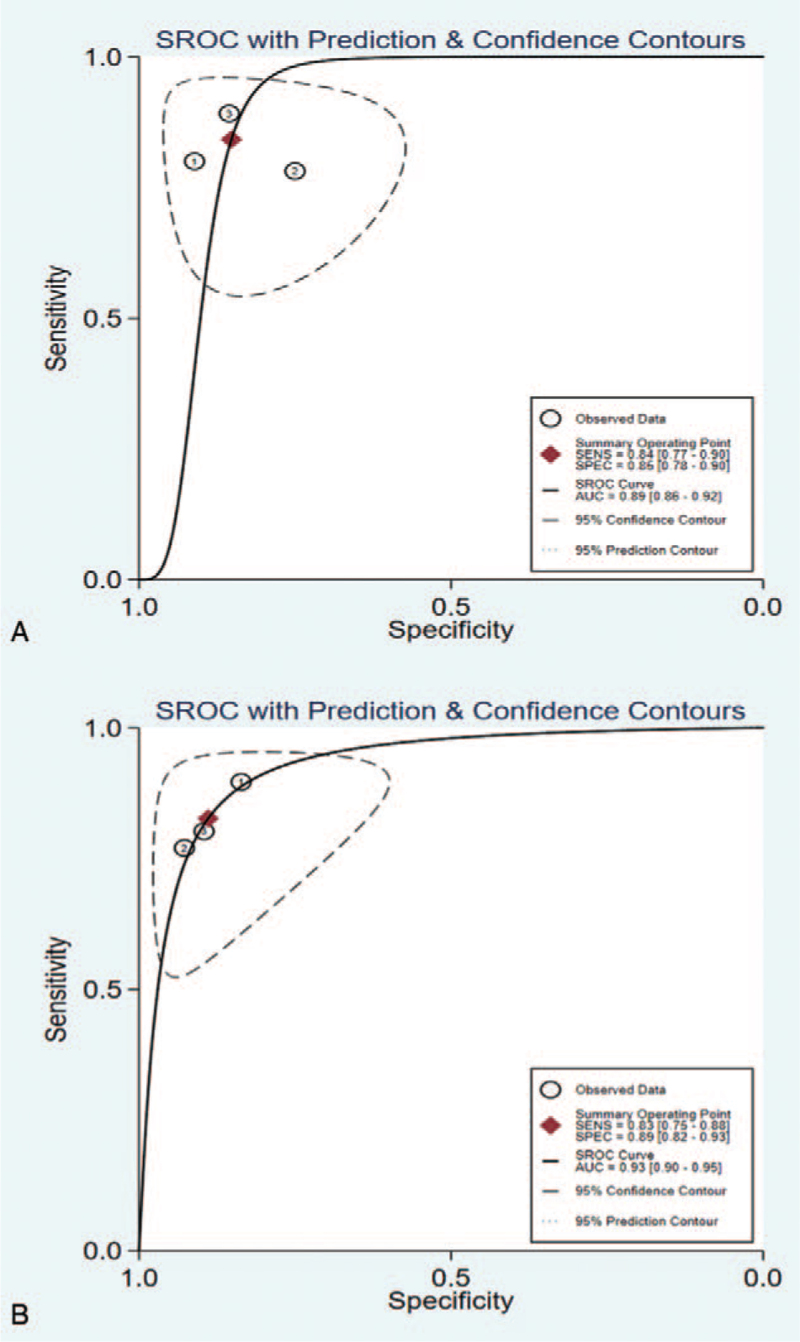
(A) The summary receiver operator characteristic (SROC) curve of ovarian cancer. (B) The SROC curve of cervical cancer.

The outcomes of pooled SEN and SPE of cervical cancer were shown in Figure 4. A fixed-effect model was applied due to the no significant heterogeneity between the groups. The summary estimates are as follows: SPE, 0.89 (95% CI 0.82–0.93); SEN, 0.83 (95% CI 0.75–0.88); besides, a summary receiver operator characteristic curve was carried out in Figure 5 and AUC was 0.93 (95% CI 0.90–0.95).

### 3.6 Publication bias

**Figure 6:**
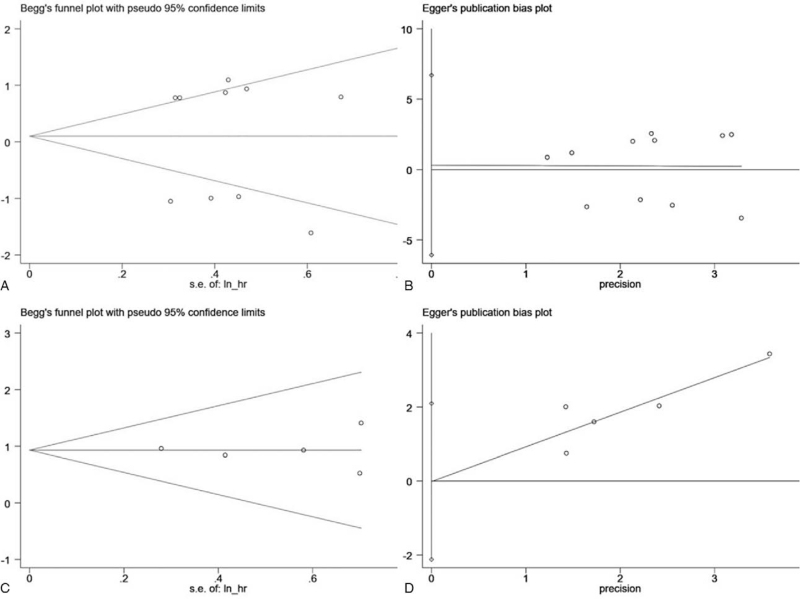
(A) Begg test for ovarian cancer oncogenic circRNAs in predicting the overall survival. (B) Egger test for ovarian cancer tumor-suppressor circRNAs in predicting the overall survival. (C) Begg test for cervical cancer oncogenic circRNAs in predicting the overall survival. (D) Egger test for cervical cancer oncogenic circRNAs in predicting the overall survival.

Judged by a Deeks funnel plot, there was no evidence of publication bias (*P* = .13) in the diagnostic analysis (see Fig. 2, Supplemental Content, which illustrates the publication bias judged by Deeks funnel plot for the diagnostic meta-analysis). Publication bias can be measured using Begg funnel plot and Egger test. A Begg funnel plot (Fig. 6A, *P* = .976) and an Egger test (Fig. 6B, *P* = .956), indicated that there was no clear publication bias in the analysis of the tumor-suppressor circRNAs in terms of OS. There was also no publication bias in the analysis of oncogenic circRNAs in the case of OS, as indicated by a Begg funnel plot (Fig. 6C, *P* = .945) and an Egger test (Fig. 6D, *P* = .900). These outcomes indicated that circRNAs are likely to be a favorable diagnostic and prognostic biomarker.

## 4 Discussion

This is the first meta-analysis explored circRNA for the diagnosis and prognosis of ovarian and cervical cancer. In terms of its mechanism and expression level, we regarded circRNAs as tumor promoters or tumor suppressors. Our outcomes demonstrated that dysregulated expression of circRNAs had a different impact on ovarian and cervical cancer survival. High expression levels of oncogenic circRNAs were significantly associated with poor prognoses both in ovarian and cervical cancer. However, elevated expression levels of tumor-suppressor circRNAs were linked with favorable survival time in ovarian cancer. In addition, the diagnostic significance of circRNAs was investigated as well. The AUC value in ovarian cancer and cervical cancer is 0.89 and 0.93, respectively. This suggesting that circRNA will be a novel biomarker in diagnosis. Besides, there was no obvious heterogeneity and publication bias performed by a SEN analysis.

Previous studies discovered circRNA can affect tumorigenesis, metastasis, and rebuilding of the tumor microenvironment. Through the study of Li et al,^[16]^ overexpression of circ100395 can inhibit the proliferation, migration, and invasion of ovarian cancer cells by regulating the miR-1228/p53/EMT axis; at the same time, Cai et al^[40]^ found that circ0000263 was significantly upregulated in cervical cancer cells and ultimately affected the expression of p53 gene. We found p53 gene was a common gene in the development of ovarian cancer and cervical cancer. According, we inferred the potential use of circRNAs to regulated p53 gene might as therapeutic targets for the treatment of ovarian and cervical cancer. Several previous meta-analyses^[41–42]^ reported circRNA has important diagnostic and prognostic value in tumor. Huang et al^[43]^ have summarized that circRNAs may act as important biomarkers for diagnosis and prognosis in diverse cancers by meta-analysis. There are few studies on ovarian and cervical cancer included in previous study. In our research, 22 studies involving 2059 patients with ovarian or cervical cancer were included, which markedly increased the statistical power and made the pooled results more credible. Additionally, the functions and mechanisms of circRNA in both cancers have been clarified.

Despite the promising data, there are some limitations to our study. First, the samples were all taken from cancerous tissues, and the diagnostic value of plasma samples was higher. More plasma samples are needed for further study. Second, all the patients in our study were selected from an Asian population, so the results may be biased. Third, the sample size in this study was small, so a larger clinical study is needed. Finally, some articles did not directly provide the survival data, so we had to estimate the HRs from Kaplan-Meier curves by the method of Parmar et al.^[32]^

## 5 Conclusions

CircRNAs have the prospect of becoming a promising biomarker for diagnosis and prognosis of ovarian and cervical cancer. Accordingly, circRNAs might be novel indicators and targets of therapy for ovarian and cervical cancer.

## Acknowledgments

The authors thank Editage (www.editage.cn) for English language editing.

## Author contributions

All authors have read and approved the manuscript.

**Conceptualization:** Fengyuan Liu; Xinrui Wu, Huixia Zhu.

**Data curation:** fengyuan liu.

**Formal analysis and investigation:** Fengyuan Liu.

**Formal analysis:** fengyuan liu.

**Funding acquisition:** Feng Wang.

**Methodology:** Fengyuan Liu; Huixia Zhu; Feng Wang.

**Project administration:** feng Wang.

**Resources:** Feng Wang.

**Supervision:** Huixia Zhu; Xinrui Wu.

**Writing – original draft preparation:** Fengyuan Liu.

**Writing – review & editing:** Feng Wang; Xinrui Wu.

## Supplementary Material

Supplemental Digital Content

## Supplementary Material

Supplemental Digital Content

## Supplementary Material

Supplemental Digital Content
