# Supplementary material for: Dysregulated expression of circular RNAs serve as diagnostic and prognostic markers in ovarian and cervical cancer: A PRISMA-compliant systematic review and meta-analysis
Source: Medicine (Baltimore). 2021 Oct 1;100(39):e27352. doi: 10.1097/MD.0000000000027352 (PMC8483828; doi:10.1097/MD.0000000000027352)

**Figure 1, Supplemental Content,** which illustrates the Study quality assessed by the QUADAS II tool.


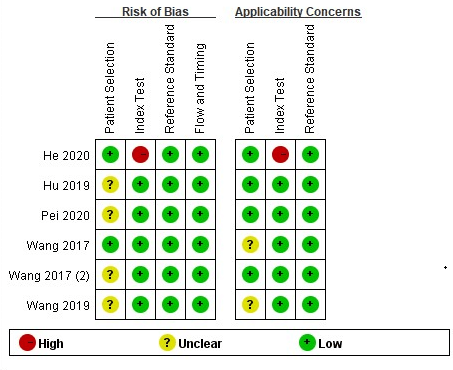

Supplement: Supplemental Digital Content [file medi-100-e27352-s001.docx]
