# Supplementary material for: Dysregulated expression of circular RNAs serve as diagnostic and prognostic markers in ovarian and cervical cancer: A PRISMA-compliant systematic review and meta-analysis
Source: Medicine (Baltimore). 2021 Oct 1;100(39):e27352. doi: 10.1097/MD.0000000000027352 (PMC8483828; doi:10.1097/MD.0000000000027352)
Supplement: Supplemental Digital Content [file medi-100-e27352-s002.docx]

**Table 3, Supplemental Content, which illustrates study quality assessed via the Newcastle-Ottawa Scale checklist.**

| Study | Selection | Comparability | Outcome | Total score |
| --- | --- | --- | --- | --- |
| Xu et al. [32] | ☆☆ | ☆☆ | ☆☆☆ | ☆☆☆☆☆☆☆ |
| Liu et al. [25] | ☆☆☆ | ☆☆ | ☆☆☆ | ☆☆☆☆☆☆☆☆ |
| Luo et al. [30] | ☆☆☆ | ☆☆ | ☆☆ | ☆☆☆☆☆☆☆ |
| Sun et al. [31] | ☆☆☆ | ☆☆ | ☆☆ | ☆☆☆☆☆☆☆ |
| Zhang et al. [33] | ☆☆☆ | ☆☆ | ☆☆☆ | ☆☆☆☆☆☆☆☆ |
| Sheng et al. [26] | ☆☆☆ | ☆☆ | ☆☆☆ | ☆☆☆☆☆☆☆ |
| Li et al. [29] | ☆☆☆ | ☆☆ | ☆☆ | ☆☆☆☆☆☆☆ |
| Chen et al. [27] | ☆☆☆ | ☆☆ | ☆☆☆ | ☆☆☆☆☆☆☆☆ |
| Zhang et al. [34] | ☆☆ | ☆☆ | ☆☆ | ☆☆☆☆☆☆ |
| Zou et al. [35] | ☆☆☆ | ☆☆ | ☆☆☆ | ☆☆☆☆☆☆☆☆ |
| Sun et al. [39] | ☆☆☆ | ☆☆ | ☆☆☆ | ☆☆☆☆☆☆☆☆ |
| He et al. [20] | ☆☆☆ | ☆☆ | ☆☆☆ | ☆☆☆☆☆☆☆ |
| Ji et al. [28] | ☆☆☆ | ☆☆ | ☆☆ | ☆☆☆☆☆☆☆ |
| Ding et al. [36] | ☆☆ | ☆☆ | ☆☆ | ☆☆☆☆☆☆ |
| Song et al. [38] | ☆☆☆ | ☆☆ | ☆☆☆ | ☆☆☆☆☆☆☆☆ |
| Hong et al. [37] | ☆☆ | ☆☆ | ☆☆ | ☆☆☆☆☆☆ |
